# Long-term response to sequential anti-HER2 therapies including trastuzumab-deruxtecan in a patient with HER2-positive metastatic breast cancer with leptomeningeal metastases: a case report and review of the literature

**DOI:** 10.3389/fonc.2023.1210873

**Published:** 2024-01-10

**Authors:** Axel de Bernardi, Thomas Bachelot, Louis Larrouquère

**Affiliations:** ^1^ Department of Medical Oncology, Centre Léon Bérard, Lyon, France; ^2^ Cancer Reseach Center of Lyon, Lyon, France

**Keywords:** leptomeningeal metastasis, breast cancer, HER2, trastuzumab-deruxtecan, tucatinib

## Abstract

The incidence of leptomeningeal metastases (LM) is increasing among breast cancer patients, but their prognosis remains dismal. Many therapeutic options are now available to treat HER2-positive (HER2+) metastatic breast cancer (MBC) involving the central nervous system (CNS). This case report illustrates a long-lasting response of more than 2 years in a patient with HER2+ MBC with LM after sequential administration of systemic and intrathecal (IT) anti-HER2 therapies and highlights that an appropriate treatment of HER2+ LM can result in durable survival.

## Introduction

Leptomeningeal metastasis (LM) corresponds to the spread of malignant cells in the subarachnoid space (leptomeninges). LM is a frequent complication of metastatic breast cancer (MBC), with high morbidity and mortality rates. Breast cancer (BC) is one of the most frequent primary tumor associated with LM (12-35%), with a median OS ranging from 3 to 4 months (1). LM is detected in approximately 10-12% of patients with BC at diagnosis (2) but is often underdiagnosed with a 20% rate of leptomeningeal involvement in autopsy series (3, 4). LM occurs in the presence of synchronous CNS metastases in 43%–52% of cases (5–7). The prevalence of HER2-low (IHC score of 1+ or 2+ with a negative FISH result) and HER2+ (IHC score of 2+ with a positive FISH result or IHC score of 3+) BM is approximately 15% and 50%, respectively (8). The HER2+ subtype seems to be more prone to developing LM, though only the triple-negative BC subtype has been consistently associated with an increased risk of LM in the literature (9, 10). Other risk factors of developing LM include a younger age, extracranial disease at diagnosis and/or a medical history of brain metastasis (BM) surgery, especially infratentorial BM (11, 12). Best supportive care remains the most appropriate strategy for patients with severe neurological impairment and when only weak therapeutic options are available. To guide treatment decision in patients with LM, clinicians can take into consideration the prognostic Curie score that integrates performance status, the number of chemotherapy (CT) regimens prior to LM diagnosis and negative hormone receptor status, and, in patients with LM from BC, the breast graded prognostic assessment (Breast-GPA) initially validated in patients with BC brain metastases (BCBM) (13). Other prognostic factors include initial response to treatment and protein levels in the cerebrospinal fluid (CSF) at diagnosis (1). However, though effective therapies are available in some cases, a treatment decision should be made as soon as possible.

The therapeutic strategy for patients with LM is based on expert opinions summarized in the European Association of Neuro-Oncology-European Society for Medical Oncology (EANO-ESMO) clinical practice guidelines published in 2017 (14). However, none of these therapeutic options increased overall survival (OS) in patients with LM. In HER2+ MBC patients, the improvement of systemic anti-HER2 targeted therapies has led to better extracranial response rates and prolonged OS. Small CNS metastases have an intact BBB which limits the penetration of drugs into the tumor, while larger CNS metastases show BBB disruption and inhomogeneity across the tumor. The incidence of LM is increasing as the CNS penetrance of systemic agents at therapeutic doses is limited by the meningeal-blood barrier.

Here, we present the case report of a patient with HER2+ BC with LM. Her therapeutic management included the sequential administration of both systemic and intra-CSF therapies with a prolonged OS over 2 years after the initial diagnostic of LM.

## Case report

In 2016, a 38-year-old female patient underwent a right mastectomy with ipsilateral sentinel nodal dissection for a pT2N1mi(sn) non-specific infiltrating ductal carcinoma (SBR grade 3, Estrogen Receptor (ER) negative, Progesterone Receptor (PR) negative, HER2 3+, positive vascular emboli). The patient received adjuvant CT (3 cycles of fluorouracil-epirubicin-cyclophosphamide, 1 cycle of trastuzumab-docetaxel followed by 6 weekly cycles of trastuzumab-paclitaxel) and adjuvant local radiation therapy. Trastuzumab injections were continued to totalize 1 year of treatment.

**Figure 1:**
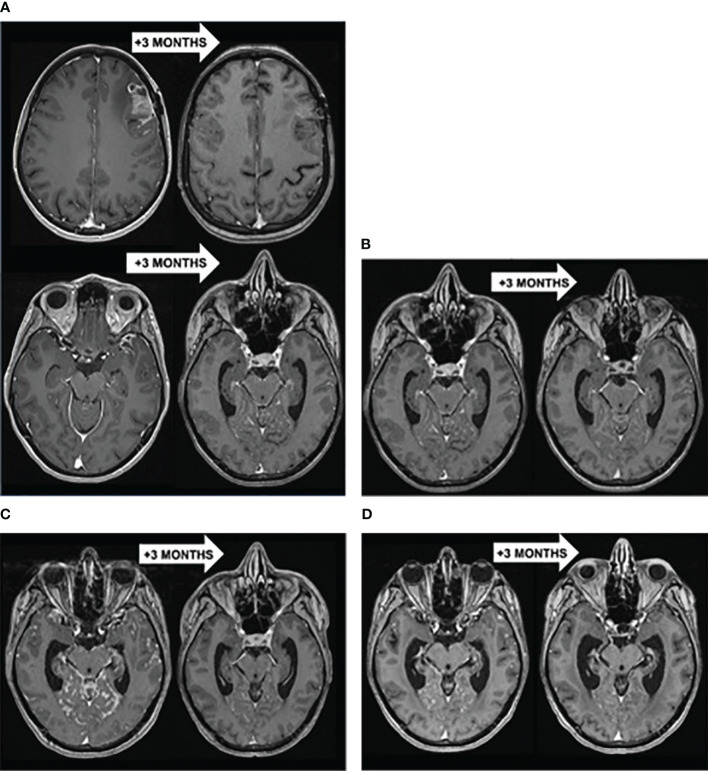
**(A)** Brain T1-WI MRI showing a PR of the left frontal lesion but progression of other LM lesions to stereotactic radiation therapy (5*6 Gy) followed by paclitaxel-trastuzumab-pertuzumab. **(B)** Brain T1-WI MRI showing a diffuse PR of LM to intra-CSF MTX (3*15 mg) followed by intra-CSF trastuzumab (150 mg) with concomitant IV pertuzumab. **(C)** Brain T1-WI MRI showing a diffuse PR of LM to WBRT (10*3 Gy) with concomitant IV trastuzumab-emtansine. **(D)** Brain T1-WI MRI showing a diffuse PR of LM to IV trastuzumab-deruxtecan.

After 2 years of follow-up, the patient was referred to an emergency department to explore headaches of rapid-onset associated with fatigue (KPS = 80%) and vomiting. Brain MRI revealed a left frontal meningeal lesion, isointense on T1-weighted imaging (T1-WI) and T2-weighted imaging (T2-WI), strongly enhanced after gadolinium injection, with multiple linear contrast-enhanced leptomeningeal lesions in the frontotemporal, peri-mesencephalic and peri-bulbar region (**Figure 1A**). There was no distant metastasis on the thoraco-abdominopelvic CT-scan. A stereotactic brain biopsy confirmed the diagnostic of LM with the same profile compared to the primary tumor (ER negative, PR negative, HER2 3+). The patient received stereotactic radiation therapy (5*6 Gy) for the left frontal lesion followed by systemic intravenous (IV) CT with paclitaxel, trastuzumab and pertuzumab. Despite a radiological partial response (PR) at 3 months on the left frontal lesion, other leptomeningeal lesions progressed, which was associated with a neurological decline (KPS = 50%, cognitive impairment, cerebellar syndrome) (**Figures 1A, B**). A lumbar puncture (LP) was performed and malignant cells were detected in the CSF. The patient was treated with dose-dense intra-CSF injections of methotrexate (MTX) (3 injections of 15 mg over a week) followed by weekly intra-CSF trastuzumab injections (150 mg) with concomitant IV pertuzumab. That therapeutic association led to a complete response (CR) in the CSF (no malignant cells found) and to a radiological PR. The patient experienced a durable neurological improvement (KPS = 80%) and could return home for 6 months (**Figure 1B**).

LM recurred with a micronodular pattern in the cerebrum, brainstem and cerebellum. A new line of systemic trastuzumab-emtansine (T-DM1) in monotherapy was initiated but showed no clinico-radiological benefit after 3 cycles (**Figure 1C**). Consequently, the patient received a whole-brain radiotherapy (WBRT) (10*3 Gy) with concomitant T-DM1, resulting in a 3.5 months-PR when the patient could stay at home (**Figure 1C**). That improvement was followed by a neurological decline (KPS = 50%) and radiological progression of LM. A new systemic treatment combining tucatinib, trastuzumab and capecitabine was initiated with a transient clinical improvement for 1.5 months (KPS = 60%).

Eventually, the LM progressed (**Figure 1D**) and a last treatment of IV trastuzumab-deruxtecan (T-DXd) resulted in a clinico-radiological PR at 3 months with stable neurologic symptoms (KPS = 50%) (**Figure 1D**). After 5.4 months, the disease progressed again and the patient died 27 months after the diagnosis of LM, resulting in an OS of 6.8 months since the initiation of T-DXd.

## Discussion

### Current management of patients with HER2+ LM

#### Diagnostic of LM

LM is a diagnostic challenge often suspected based on the conjunction of clinical evaluation, radiological and CSF findings.

The clinical presentation consists in raised intracranial pressure, meningeal irritation and multifocal neurologic signs of rapid onset such as leg weakness, cauda equina syndrome or diplopia (15, 16).

Craniospinal MRI has a high sensitivity rate that can vary between 66% and 98%, depending on the series (17–20). Nevertheless, MRI sensitivity can greatly vary depending on the experience of the neuroradiologist and the use of appropriate sequences (contrast-enhanced T2 FLAIR, three-dimensional T1 black-blood fast spin-echo imaging) (21). Pathologic linear or nodular meningeal contrast-enhancements are classically visualized on gadolinium-injected T1-WI and localized on the cortical surface, gyri and sulci, cerebellar folia, the ventral surface of the brainstem and the spinal cord (22–24). Cranial nerves and spinal roots can also be pathologically enhanced.

Still, LM sometimes occurs without radiological evidence of LM. Given the very high sensitivity and specificity of the detection of malignant cells in the CSF compared to neuroimaging (17, 19, 25, 26), it is the gold standard for LM diagnosis and should be performed at diagnosis. Malignant cells are detected in approximately 66-90% of patients with LM from MBC (27). CSF profile might also include elevated protein rate (60-80%) and lymphocytic pleocytosis (50-60%). Although not specific to LM, increased lactate dehydrogenase (LDH) levels can be helpful in evaluating LM response follow-up (28). Increased Cancer antigen 15-3 (CA 15-3) CSF levels (> 3.0 IU/mL) can also be used to detect LM in MBC, with a sensitivity of 80% and a specificity of 70% (29). If the clinical suspicion of LM is high, LP should be repeated with a minimum CSF volume of 10.5 mL and be processed rapidly to minimize false-negative rates (26, 30). Still, CSF cytology can remain negative even in the presence of radiological features of LM (15, 30). A negative cytology should not prevent treatment initiation when symptoms and MRI are highly suggestive of LM. The diagnostic value of circulating tumor cell detection in the CSF is under investigation but is currently not routinely used (31, 32).

The EANO-ESMO clinical practice guidelines 2017 introduced a classification of LM based on CSF and clinico-radiological features to provide support for decision-making (14). Type I LM corresponds to a positive CSF cytology or biopsy, and type II to clinical findings and neuroimaging only. Each type is further classified into several subtypes according to clinico-radiological features: type A refers to linear disease only, type B to nodular disease only, type C to the combination of both and type D to a normal MRI. Other criteria such as the presence of synchronous or metachronous BM, pathology and molecular tumor profile (initial diagnosis/at relapse), prior treatments (focal and/or systemic) and the evolution of the extra-CNS disease are taken into consideration.

## Available treatment options for patients with HER2+ LM from BC

Patients with LM have seldom been included in large prospective BM trials, mainly due to their dismal prognosis. Consequently, data to guide therapeutic decision are lacking in that population. Therapeutic strategies are usually extrapolated from experience in patients with BCBM or even MBC. Treatment modalities include intrathecal (IT) chemotherapy (CT), WBRT and systemic CT (1) and their combination should be discussed in multidisciplinary tumor boards. The therapeutic options discussed hereafter are based on retrospective data and expert consensus, with a low level of evidence (**Table 1**).

**Table 1:** Summary of published data on patients with leptomeningeal metastases from HER2-positive breast cancer with more than 5 patients and median overall survival available.

Author	Number of patients	mOS	mCNS-PFS
Zagouri et al. (33) Intrathecal trastuzumab (pooled analysis, 13 articles)	17	13.5 months	7.5 months
Bonneau et al. (34) Intrathecal trastuzumab (phase 1 study)	16	7.3 months	–
Figura et al. (35) Intrathecal trastuzumab	18	13.2 months	5.4 months
Zagouri et al. (36) Intrathecal trastuzumab (pooled analysis, 24 articles)	58	13.2 months	5.2 months
Carausu (13) Intrathecal therapy (methotrexate, cytarabine, or thiotepa)	47	5.6 months	–
Oberkampf et al. (37) Intrathecal trastuzumab (150 mg/week)	19	7.9 months	5.9 months
Kumthekar et al. (38) Intrathecal trastuzumab (80 mg twice a week)	23	10.5 months	2.8 months
Murthy et al. (39) Oral tucatinib and trastuzumab IV and oral capecitabine	17	11.9 months	6.9 months
Pellegrino et al. (40) Oral neratinib and oral capecitabine	10	10 months	4 months
Alder et al. (41) Trastuzumab deruxtecan IV	8	10.4 months	–
Niikura et al. (42) Trastuzumab deruxtecan IV	19	10.4 months	–

mOS: median overall survival; mCNS-PFS: median central nervous system progression-free survival; IV: Intravenous.

### Intrathecal therapies for patients with HER2+ LM

Intra-CSF pharmacotherapies are widely used in patients with LM across Europe, and should be restricted to patients with a life expectancy ≥ 1 month (14, 43). There are two routes of administration: repeated LP or intraventricular catheter, like Ommaya reservoirs. Four agents are classically used for intra-CSF injections: MTX, cytarabine (including liposomal cytarabine), topotecan and thiotepa (44, 45). There is no OS benefit of one agent over another and the combination of intra-CSF CT is not superior to monotherapy (46–48). The secondary analysis of a randomized trial showed that patients with LM (n = 100, 36 cases of primary BC) treated with intraventricular MTX had a longer progression-free survival (PFS) compared to lumbar administration (49). Ommaya reservoirs have a low complication rate (< 7.4% in the literature) with a uniform distribution in the CNS and avoid discomfort and post-procedural complications linked to repeated LP.

The EANO-ESMO 2017 classification provided a rationale for using intra-CSF pharmacotherapy in patients with linear or ependymal MRI contrast-enhancement and/or a positive cytology. Intra-CSF pharmacotherapy is recommended in patients with a positive CSF cytology (*i.e.* all type I LM), irrespective of their MRI presentation. In patients with a negative CSF cytology (type II LM), intra-CSF pharmacotherapy can also be considered in case of linear contrast enhancement (type IIA) or linear and nodular disease (type IIC). Conversely, a nodular meningeal disease alone (type IIB) predicts a poor CNS penetration of intra-CSF pharmacotherapy and can even increase the toxicity of chemotherapeutic agents due to CSF blocked flow. CSF flow studies can be done in this situation and radiation can be given to the site of the block to alleviate it.

Only 6 randomized trials prospectively explored the benefit of intra-CSF chemotherapy in LM, with contrasting results (46–48, 50–52). Two trials specifically assessed the efficacy of adding intra-CSF chemotherapy to systemic CT in patients with LM from BC. Boogerd et al. selectively enrolled patients with LM from BC and compared the combination of intra-CSF MTX with systemic CT (n = 17) to systemic CT alone (n = 18) (50). The study failed to demonstrate a clear benefit of adding intra-CSF MTX with similar response rates (59% versus 67%) and a shorter median OS (mOS) of 18.3 weeks in the experimental arm (versus 30.3 weeks, 95% CI: 5.5-34.3 weeks, p = 0.32). The poor prognosis observed in the experimental arm could partly be explained by a 18% Ommaya reservoirs revision rate and a higher rate of neurological complications (47% versus 6% in the control group, p = 0.0072). The DEPOSEIN open-label phase III trial evaluated the efficacy of adding intra-CSF liposomal cytarabine to systemic CT alone in 73 patients with LM from BC (52). The patients treated with the experimental treatment had a longer PFS related to LM (LM-PFS) of 3.8 months versus 2.2 months in the control arm (HR = 0.61, 95% CI: 0.38-0.98, p = 0.04) and a mOS of 7.3 months (95% CI: 3.9-9.6) versus 4.0 months (95% CI: 2.2-6.3) in the control group. The control group had a higher rate of HER2+ tumors (24% versus 6% in the experimental group) and most frequently received anti-HER2 therapies (n = 7 versus n = 2 in the experimental group).

Based on the poor CNS penetrance of systemic trastuzumab with a serum-to-CSF rate of 1/420 (53) and the concordant HER2 status between CSF cancer cells and primary tumor, case reports of long-responders (> 2 years) to intra-CSF trastuzumab were published (53, 54).

A first prospective multi-institutional phase I/II dose escalation trial was done to evaluate intra-CSF trastuzumab at a dose of 80 mg (2x/week for a month, then 1x/week for a month, then 1x/2 weeks) in 34 patients with LM from BC (55). The mOS was 12.1 months (95% CI: 4.3-19.6) with a clinical benefit in 69% of cases. Another phase I dose-escalation study published by Bonneau et al. assessed intra-CSF trastuzumab (150 mg weekly) in 19 patients with HER2+ LM from BC (34). At a dose of 150 mg, the mean trastuzumab CSF residual concentration was 27.88 mg/L, similar to the optimal inhibition concentration (30 mg/L). 3 patients had a clinical response, 7 were stable and 4 progressed with no reported dose-limiting toxicities. In a pooled analysis of 17 patients from 13 articles, the administration of intra-CSF trastuzumab was safe with a median PFS (mPFS) of 13.5 months and a median CNS-PFS of 7.5 months (33). A prospective trial compared the efficacy of intra-CSF trastuzumab (n = 18), single-agent intra-CSF CT (n = 15) or WBRT alone (n = 23) in 56 patients with HER2+ BC with LM (35). The CNS-PFS at 6 months was 44% with intra-CSF trastuzumab (versus 18% and 26% in the intra-CSF CT and WBRT groups) with a 1-year OS of 54% (versus 10%, and 19% in the intra-CSF CT and WBRT groups). More recently, a meta-analysis that included 58 patients with HER2+ LM treated with intra-CSF trastuzumab reported a shorter CNS-PFS of 5.2 months but a mOS of 13.2 months from intra-CSF trastuzumab initiation (36). Recently, a phase I/II prospective trial that enrolled 23 patients with HER2+ LM treated with IT trastuzumab was performed. At a dose of 80 mg twice weekly, the mOS was 10.5 months (95% CI 5.2-20.9) (38). A single-arm, non-randomized phase I/II trial (NCT04588545) will assess the efficacy of radiotherapy (RT) (WBRT or focal RT) followed by intra-CSF trastuzumab/pertuzumab (with pertuzumab dose-escalation) in HER2+ LM (56).

### Systemic treatments for patients with HER2+ LM

Case reports and retrospective series suggested some efficacy of systemic treatments in LM from MBC, but very few prospective trials are available to guide therapeutic decision. Consequently, guidelines for systemic treatments are extrapolated from the management of HER2+ BM (14, 57).

There is one case report of HER2+ LM response to T-DM1 published in the literature (58). The patient had BM and LM and received T-DM1 with concomitant WBRT after a first line of pertuzumab, trastuzumab and docetaxel. The patient experienced a CR after 3 cycles with a favorable safety profile and long-lasting CNS control > 13 months. In 2020, Higashiyamaa et al. published the case of a patient with heavily pretreated HER2+ BC with metastases to the CNS and liver (59). The diagnostic of LM was suspected based on clinical and MRI features, CSF cytology was not documented. The patient had an objective radiological improvement of LM lesions after 9 cycles of dose-adapted trastuzumab-deruxtecan (T-DXd). More recently, Alder et al. published a series of 8 patients with heavily pretreated HER2+ MBC and progressing LM treated with T-DXd. All the patients experienced a clinical benefit from T-DXd and 4 patients had a PR (41). We can cite the case of a patient with HER2-negative MBC (60) who experienced BM and LM treated with lapatinib (61). The patient had an objective neurological improvement during 9 months and experienced a rapid neurological decline when lapatinib was discontinued. One of the metastatic skin tumors was HER2 2+ (FISH -), which could suggest receptor conversion from HER2- to HER2+ in LM.

The first retrospective studies of patients with LM suggested some activity of systemic CT (50, 62, 63). In 2015, the case series of Chahal et al. evaluated IV thiotepa (40 mg/m^2^ every 21 days) in 13 patients with LM secondary to BM (64). 4 patients displayed a PR, 3 SD and 6 PD with a 69% 6-month survival rate and a 31% 1-year survival rate. The recent series of Pellegrino et al. evaluated the activity of Neratinib in association with Capecitabine in 10 patients with LM from heavily pretreated HER2+ BC (40). Patients experienced a 6-month OS of 60%, and a mOS of 10 months (95% CI: 2.00-17.0). 3-month intracranial PFS (IC-PFS) was 60% with a median IC-PFS of 4 months (95% CI: 2.00-6.0) and a median duration of neurological response of 6.5 months.

In 2015, Wu et al. assessed in a pilot study the benefit of bevacizumab combined with etoposide and cisplatin (BEEP) in 8 patients with LM from BC (65). The CNS-overall response rate (ORR) was 60% with a mOS of 4.7 months (95% CI: 0.3-9.0) and a CNS-PFS of 4.7 months (95% CI 0-10.5). The single-arm phase II trial published by Brastianos et al. assessed pembrolizumab efficacy in patients with solid tumor malignancies and LM (66). 15 patients had LM from BC in the cohort (83.3%), and 7 patients (35%) were HER2+ with a mOS of 4.4 months (90% CI: 1–6.8). Kumthekar et al. published an open-label phase II study in 28 patients with LM secondary to BM from BC treated with ANG1005 (600 mg/m^2^ every 3 weeks) (67). In the subset of patients with LM, 79% had intracranial disease control and mOS of 8 months (95% CI, 5.4-9.4), with a reasonable safety profile.

An ongoing phase II non-randomized study (NCT03501979) will test the safety and efficacy of the HER2CLIMB regimen (tucatinib, trastuzumab, capecitabine) in patients with HER2+ BC with LM, with encouraging tucatinib CSF concentration levels in the CSF pharmacokinetic analysis (68). A phase III randomized trial (NCT03613181) will compare ANG1005 to the physician’s best choice in patients with pretreated BCBM and newly diagnosed HER2-negative LM from BC.

In summary, the modification of systemic agents should be considered in case of LM diagnosis and take into account the primary tumor histology, molecular profile and previous systemic treatment lines.

### Radiotherapy for patients with HER2+ LM

Historically, RT was the treatment of choice for LM but no prospective trial assessed the efficacy and safety of WBRT or craniospinal irradiation (CSI) alone. Only one phase II trial tested the combination of intra-CSF MTX with concomitant involved-field radiotherapy (IF-RT) across LM from various cancer subtypes (69). Among the 59 patients included, 11 (19%) were treated for BC with a mOS of 5.4 months (unspecified HER2 status). The retrospectives series of WBRT alone in patients with LM showed no survival benefit (70–74). However, WBRT does provide symptomatic relief in case of hydrocephalus or seizures. WBRT alone (30 Gy in 10 fractions or 20 Gy in 5 fractions) is used in patients with synchronous BM, symptomatic linear disease or extensive nodular LM (14, 75) and is the treatment of choice in patients with a poor KPS without good systemic treatment options.

The neurotoxicity of concomitant CNS RT and intra-CSF CT was mostly evaluated in hematology studies. In the series of Kim et al., 80 patients with CNS lymphoma or leukemia received intra-CSF MTX in addition to WBRT and 63 patients received intra-CSF MTX alone. Leukoencephalopathy developed in 60 (75%) and 35 patients (55%), respectively (76). Even though the rate of leukoencephalopathy was higher in the combination group, it did not reach significance levels in univariate analysis; there was no comparison between treatment sequences (WBRT-intra-CSF MTX versus intra-CSF MTX-WBRT). A safety window should be observed between the end of WBRT and the beginning of intra-CSF injections to avoid radio-sensitization and high-grade MTX-induced neurotoxicity (77), as high as 20% when WBRT and intra-CSF MTX are combined (69).

Conventional photon craniospinal irradiation (CSI) should be avoided given the high risk of RT-induced bone marrow toxicity, enteritis and mucositis and the limited impact on clinical outcome. CSI could be an option in selected patients with limited extra-CNS disease and good KPS. Recently, a phase I clinical trial evaluated hypofractionated proton CSI (30 Gy in 10 fractions) alone using proton therapy in patients with LM (7/24 patients had BC, with 2 patients with HER2+ LM) with a better tolerability and some durable responses (4 patients had a CNS-PFS >12 months) (78).

Focal RT (single fractions or fractionated regimens) can be discussed in symptomatic type IIB LM prior to intra-CSF CT to restore a normal CSF flow pattern, improve delivery of intra-CSF treatment and limit the risk of toxicity. In the retrospective cohort of Wolf et al., 16 patients with focal LM (n = 5 (31%) from BC) received stereotactic radiosurgery (SRS) with a median dose of 16 Gy (79). 14 patients (87,5%) were actively treated with CT, targeted therapy or immunotherapy at the time of SRS. 5 patients experienced a SD and 8 patients a PR. The mOS from SRS was 10 months. 6 of the 7 patients with disease recurrence underwent a salvage WBRT (median time of 6 months), suggesting a delay of WBRT after SRS in some patients. Focal RT should also be considered in the presence of symptomatic cranial nerve impairment, cauda equina syndrome or skull base involvement.

## Extrapolation of systemic treatments from HER2+ BM

### Systemic *therapeutic options* in *patients* with HER2+ CNS *metastases*

Currently, there is no standard-of-care for HER2+ BC with LM. Large prospective trials excluded patients with LM and intracranial response rates of systemic anti-HER2 therapies and CT, such as capecitabine in HER2+ BC with LM, are extrapolated from the retrospective and prospective characterization of patients with BC with BM (57).

Trastuzumab is a systemic humanized anti-HER2 IgG1 monoclonal antibody widely prescribed in HER2+ MBC. Despite a lack of prospective trials assessing the specific impact of trastuzumab in HER2+ CNS disease, some studies suggest a clinical benefit. A retrospective analysis showed that patients that received trastuzumab for newly diagnosed HER2+ BM had a more favorable mOS of 11.9 months (versus 3 months without trastuzumab) (80). The prospective, observational registHER study confirmed a prolonged OS of 17.5 months in patients with newly diagnosed HER2+ CNS metastases treated with trastuzumab (versus 3.8 months without trastuzumab) (81). Trastuzumab seems to delay the onset of CNS metastases in HER2+ MBC. Intra-CSF cancer cells have a conserved HER2 status compared to the primary tumor in 94% of cases (54); consequently, the combination of systemic anti-HER2 therapies with CT should always be considered in this population.

Patients with CNS metastases were excluded from the phase III CLEOPATRA trial that assessed the addition of pertuzumab to docetaxel and trastuzumab for first-line treatment of HER2+ MBC (82). In an exploratory analysis, Swain et al. evaluated the incidence and time to development of CNS metastases in patients from CLEOPATRA (83). The incidence of CNS metastases was comparable between treatment arms (12.6% versus 13.7%) but the onset of CNS metastases was delayed in the pertuzumab group (15.0 versus 11.9 months, HR = 0.58, 95% CI: 0.39-0.85) and associated with a better mOS (34.4 versus 26.3 months). Patients with stable CNS metastases were included in the PERUSE trial, which confirmed paclitaxel as a more tolerable alternative to docetaxel in combination with trastuzumab and pertuzumab with similar mPFS (19.2-23.2 versus 18.7 months) and mOS (64.0-70.9 versus 57.1 months) compared to CLEOPATRA (84, 85). More recently, the PATRICIA phase II study assessed the efficacy of high-dose trastuzumab (6 mg/kg weekly) in combination with pertuzumab in 39 patients with progressive HER2+ CNS metastases after prior RT (86). Despite a low CNS ORR of 11% (95% CI: 3-25), a significant proportion of patients experienced a clinical benefit at 4 (68%) and 6 months (51%). Notably, 2 patients had an ongoing intracranial and extracranial disease stabilization for > 2 years. Patients with LM were excluded.

In the second-line setting, T-DM1 is approved for patients with HER2+ MBC. T-DM1 is an antibody drug conjugate (ADC) composed of trastuzumab covalently conjugated with the microtubule-inhibitory agent DM1 (derivative of maytansine) by means of a stable linker (87). ADCs allow targeted intracellular delivery of highly cytotoxic agents to cancer cells and decrease their overall toxicity profile. The efficacy of T-DM1 was compared to lapatinib in combination with capecitabine (L+C) in patients with HER2+ locally advanced breast cancer (LABC) or MBC previously treated with trastuzumab and a taxane in the phase III EMILIA trial (88). Patients with active BM were excluded from EMILIA but 19.8% of the patients included in the trial had stable CNS metastases. Krop et al. published in 2015 an exploratory analysis of patients with CNS metastasis included in EMILIA (89). The mOS of patients with CNS metastasis at baseline was improved in the T-DM1 arm compared to L+C (26.8 versus 12.9 months, HR = 0.38; p = 0.008) with similar PFS (5.9 vs 5.7 months). Patients previously treated with lapatinib were excluded from EMILIA. Consequently, the phase III TH3RESA trial assessed the efficacy of T-DM1 compared to the best physician’s choice in patients with HER2+ MBC who received trastuzumab- and lapatinib-based treatments in previous lines (90). 67 patients (11.1%) included in TH3RESA had asymptomatic or treated BM (N=40 in the T-DM1 arm, N=27 in the physician’s choice arm) with a mPFS of 6.2 months in the T-DM1 group (versus 3.3 months in the physician’s choice group). After the approval of T-DM1, multiple retrospective series reported an ORR ranging between 20% and 44% in patients with HER2+ CNS metastases (91–94).

After T-DM1, subsequent treatment lines are not uniformly codified. Due to the development of resistance and dose-limiting toxicities with T-DM1 or concomitant SRS (95, 96), other HER2-targeted therapies such as T-DXd were developed. T-DXd is an ADC composed of trastuzumab, a tetrapeptide-based cleavable linker and a topoisomerase I inhibitor, exatecan derivative. T-DXd is a more efficient and specific anti-HER2 ADC compared to T-DM1 due to its linker cleaved by tumor enzymes (cathepsins B and L), higher drug-to-antibody ratio (97) and a bystander killing effect that also targets surrounding cancer cells with heterogenic HER2 expression (98). Based on the results of the phase II DESTINY-Breast01 trial (99), T-DXd was approved in second line for HER2+ MBC after one line of anti-HER2-based regimens. Patients with untreated or symptomatic CNS metastases were excluded, but 24 patients with treated and/or asymptomatic BM were included. Jerusalem et al. reported a 58.3% ORR in that subgroup with a mPFS of 18.1 months (95% CI: 6.7-18.1) (100). A subgroup analysis of 82 patients with HER2+ BM from the DESTINY-Breast03 trial showed an improved mPFS of 15 months in the T-DXd group (versus 3 months in the T-DM1 group, HR = 0.25, 95% CI: 0.31-0.45) and a 63.9% IC-ORR (versus 33.4% in the T-DM1 group) (101). In a recently published update of the DESTINY-Breast03 trial, the mOS was not reached (95% CI: 40.5 months–N/A) in the T-DXd group and was not reached (34.0 months–N/A) in the T-DM1 group (HR = 0,64, 95% CI: 0,47-0,87, p = 0.0037) (102). That OS benefit was observed across all the subgroups analyzed, including those with baseline BM. The single-arm phase II DEBBRAH trial (NCT04420598) assessed the efficacy of T-DXd (5.4 mg/kg) in patients with pretreated HER2+ or HER2-low stable (n = 8, cohort 1), untreated (n = 4, cohort 2), or progressing BCBM after local therapy (n = 9, cohort 3) (103). The first results reported an overall IC-ORR of 46.2% (95% CI: 19.2-74.9) in patients with active BMs (cohorts 2 and 3). Additional results are expected in the population of patients with LM (103). The ongoing multicentric single-arm phase II TUXEDO-4 trial (NCT06048718) will analyze the efficacy of T-DXd in patients with HER2-low BC with active BM.

Finally, the recent prospective, open-label, single-arm, phase II TUXEDO-1 trial evaluated the efficacy of T-DXd in patients with newly diagnosed untreated HER2+ BM or progressive BM after previous local therapy or previous exposure to trastuzumab and pertuzumab (104). Among the 15 patients enrolled, 2 had a CR, 9 had a PR and 3 had a SD with a best IC-ORR rate of 73.3% (95% CI: 48.1-89.1%). The median PFS was 14 months (95% CI 11.0 months to not recorded) and median OS was not reached at a median follow-up of 12 months.

Data on response to T-DXd in patients with HER2+ BC with LM were lacking until very recently. The study populations of the DESTINY-Breast01 and DESTINY-Breast03 studies did not include patients with active BM and/or LM. The multicentric, retrospective ROSET-BM study assessed the benefit of T-DXd in 104 patients with HER2+ MBC with BM and/or LM (105). IC-ORR was 62.7% in that cohort with a median IC-PFS of 16.1 months (95% CI: 12.2–N/A). Among the 19 patients with LM, the 1-year PFS and OS rates were 60.7% (95% CI: 34,5–79,1) and 87.1% (95%CI, 57.3–96.6), respectively. Alder et al. published a series of 8 patients with heavily pretreated HER2+ MBC and progressing LM treated with T-DXd. All the patients experienced a clinical benefit from T-DXd and 4 patients had a PR (41).

In subsequent lines, anti-HER2 TKI can cross the BBB with an IC-ORR ranging between 47% and 66% (106–108). The HER2CLIMB study evaluated the combination of tucatinib, capecitabine and trastuzumab for heavily pretreated patients with HER2+ MBC. Supported by the preliminary efficacy data of the phase I trial, the HER2CLIMB study included 291 patients (47.5%) with untreated or previously treated progressing BM at baseline (109). The mPFS of patients with BM was 7.6 months (95% CI: 6.2-9.5) in the experimental arm (versus 5.4 months in the placebo group, 95% CI: 4.1-5.7). Interestingly, that benefit was similar in a subgroup analysis of patients with active BM (HR = 0.49, 95% CI: 0.30-0.80). At a median OS follow-up of 29.6 months, mOS was 24.7 months for the tucatinib combination group versus 19.2 months in the placebo group (HR = 0.73, 95% CI: 0.59-0.90, p = 0.004) (110). In the updated exploratory analysis of the HER2CLIMB trial published in 2023 with an additional follow-up of 15.6 months, the median CNS-PFS was 9.6 months in the tucatinib-combination group compared to 5.6 months in the placebo group for patients with active BMs (95% CI: 7.6-11.1 vs 2.9-5.6) and the risk of developing new brain lesions was reduced by 45.1% in the tucatinib-combination group (HR = 0.55, 95% CI: 0.36-0.85, p = 0.006) (111). Yan et al. published the case of a patient resistant to neratinib and capecitabine that demonstrated a significant response of LM to tucatinib (112). In the TBCR049 trial, tucatinib was detectable in CSF within 2 hours after drug administration, with concentrations ranging from 0.57 to 25 ng/mL and CSF-to-plasma ratios of 0.83 (68). The NALA trial compared the activity of lapatinib (L+C) and neratinib (N+C) in combination with capecitabine in HER2+ MBC (113). 101 patients (16%) had asymptomatic or stable BM. The N+C group displayed an improved mPFS of 8.8 months compared to L+C in the overall population (versus 6.6 months, HR = 0.76, 95% CI: 0.63-0.93). The incidence of intervention for CNS disease was lower in the N+C group versus L+C group, suggesting a delayed CNS metastasis onset. Hence, a combination of L+C or N+C is an interesting option to avoid infusion therapies in patients with HER2+ BM previously treated with at least two anti-HER2 regimens.

## Conclusion

Despite the dismal prognosis classically associated with BC with LM, this case report highlights that long-term responses are possible with the sequential administration of anti-HER2 targeted therapies combined with local treatments. According to the recommendations of the ASCO Eligibility Criteria Working Group, future prospective trials should include patients with CNS metastases, including LM, to assess the CNS efficacy of new treatments and optimize treatment sequencing.

## Author contributions

AdB and LL designed and edited the text, table and figure as well as their legends under the supervision of TB. All authors provided scientific advice and critically revised the manuscript. We confirm that all coauthors have contributed to the generation of the manuscript and have given final approval of the version to be published.
